# Supplementary material for: Integrated genetic and epigenetic analysis of myxofibrosarcoma
Source: Nat Commun. 2018 Jul 17;9:2765. doi: 10.1038/s41467-018-03891-9 (PMC6050269; doi:10.1038/s41467-018-03891-9)
Supplement: Supplementary file 1 — Supplementary Information [file 41467_2018_3891_MOESM1_ESM.pdf]

Supplementary Information

Integrated genetic and epigenetic analysis of myxofibrosarcoma

Ogura et al.

1. **Supplementary Figures 1-8**
2. **Supplementary Tables 1-3**

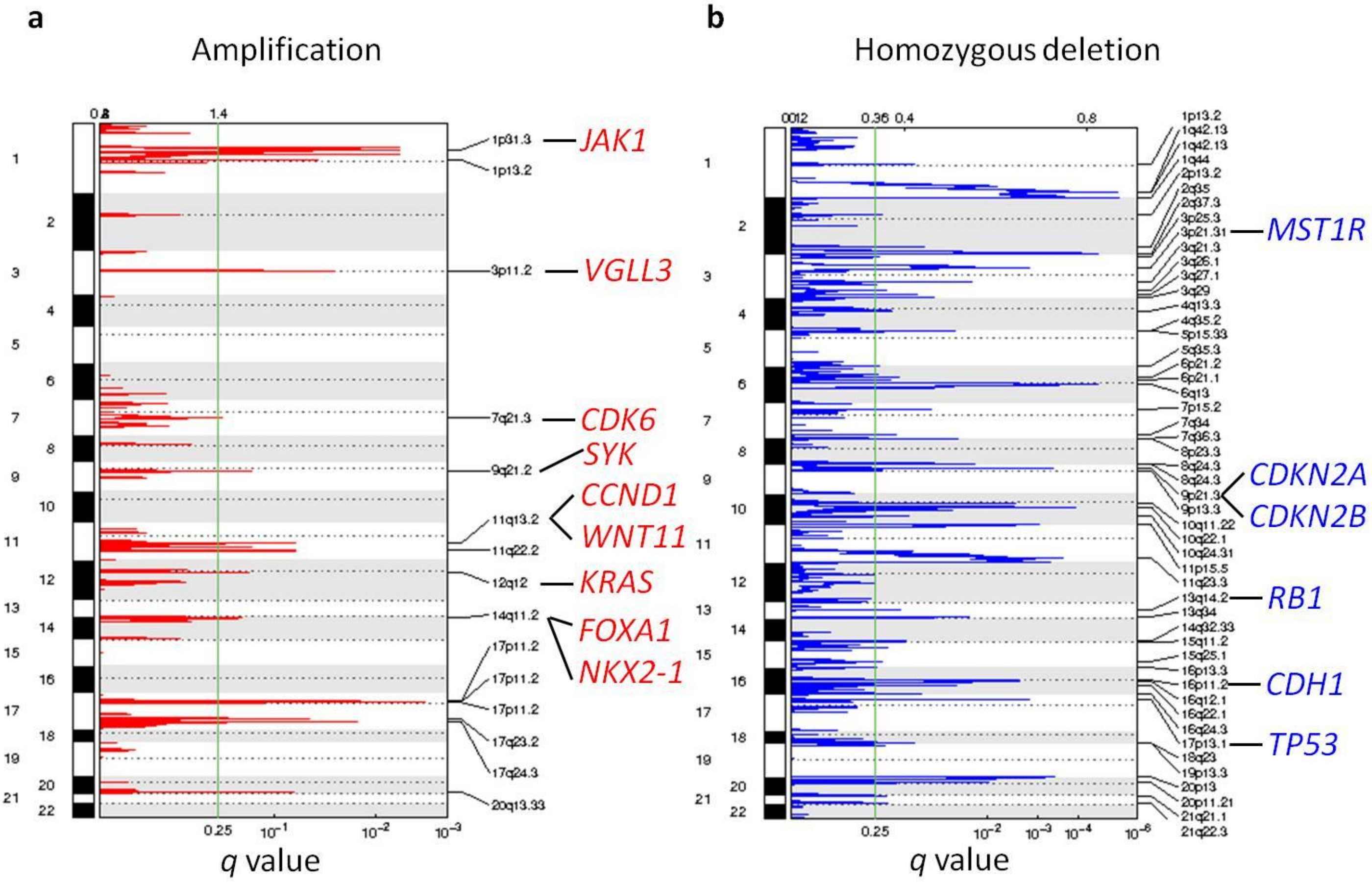

Supplementary Figure 1. Significant copy number alterations in 41 MFSs
Regions showing statistically significant focal amplifications (a) and homozygous deletions (b) detected using the GISTIC 2.0 algorithm. For each *q*-value peak, putative gene targets are listed.

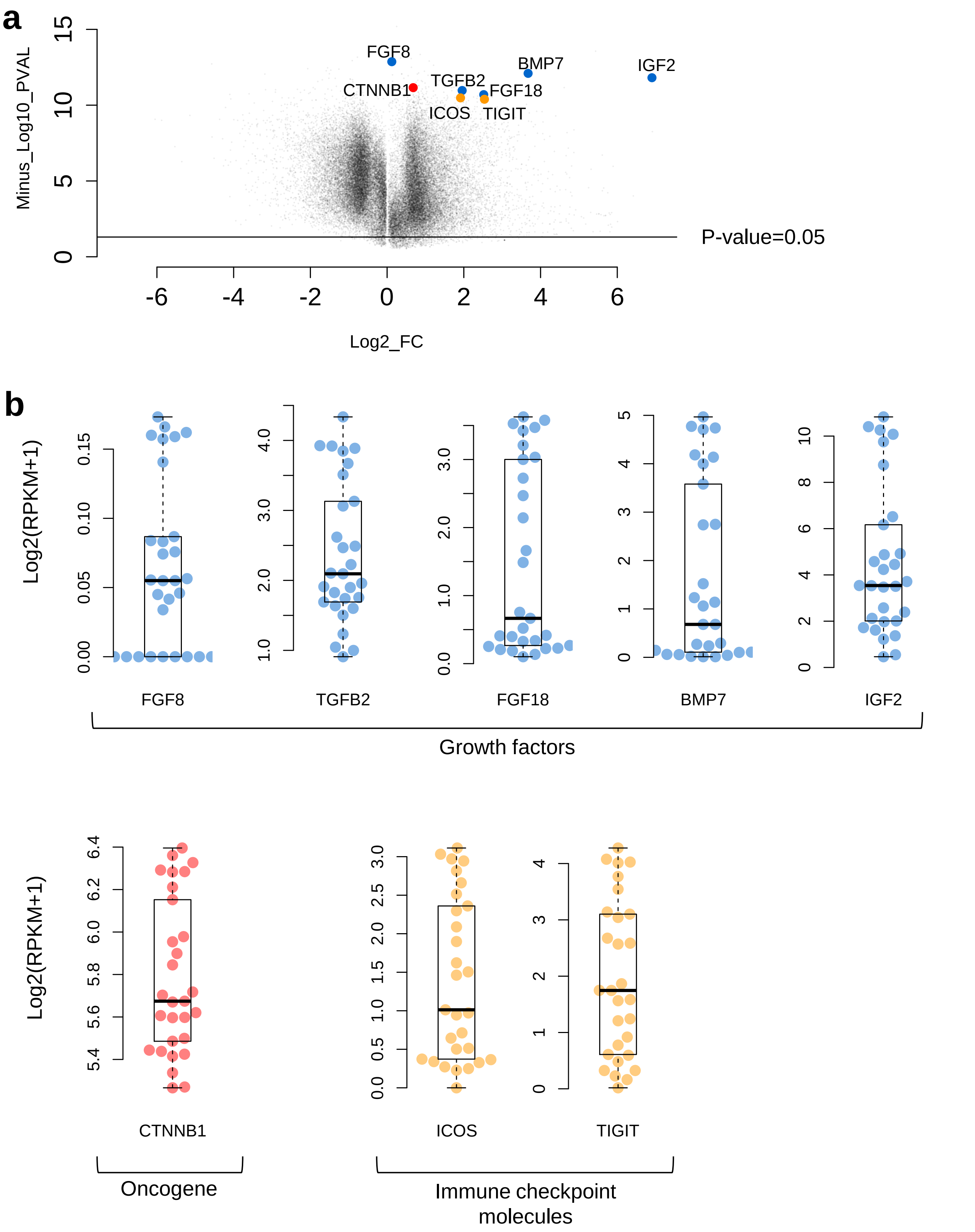

Supplementary Figure 2. Significantly up-regulated genes among 29 MFSs

(a) Distribution of significantly up-regulated genes. Interesting molecules with the top-ranked genes were shown: growth factors (*FGF8*, *BMP7*, *IGF2*, *TGFB2*, and *FGF18*), oncogenes (*CTNNB1*), and immune-related molecules (*ICOS* and *TIGIT*).

(b) Distribution of 29 MFSs in each gene.

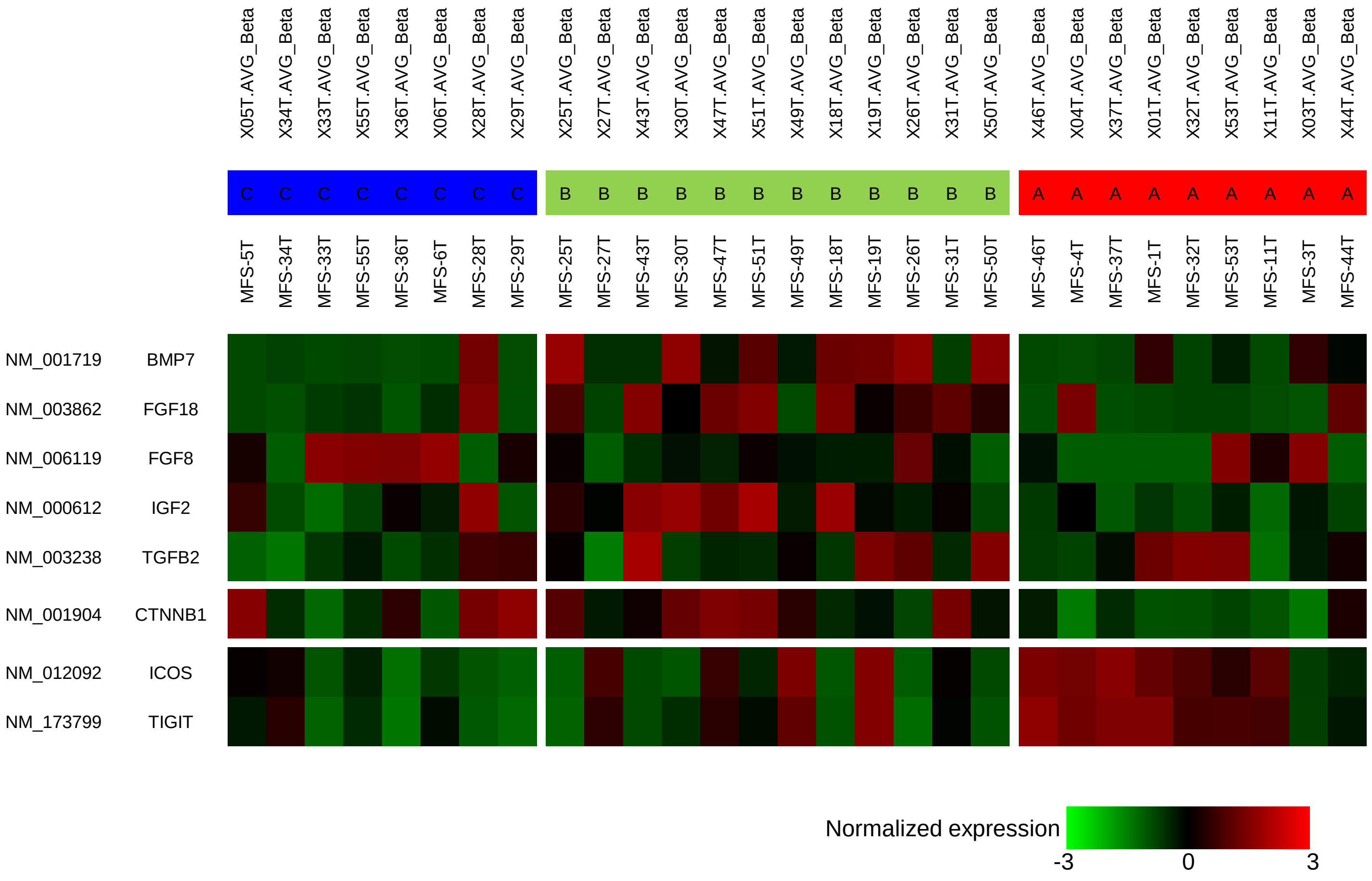

Supplementary Figure 3. Relation between methylation clusters and expression of significantly up-regulated genes

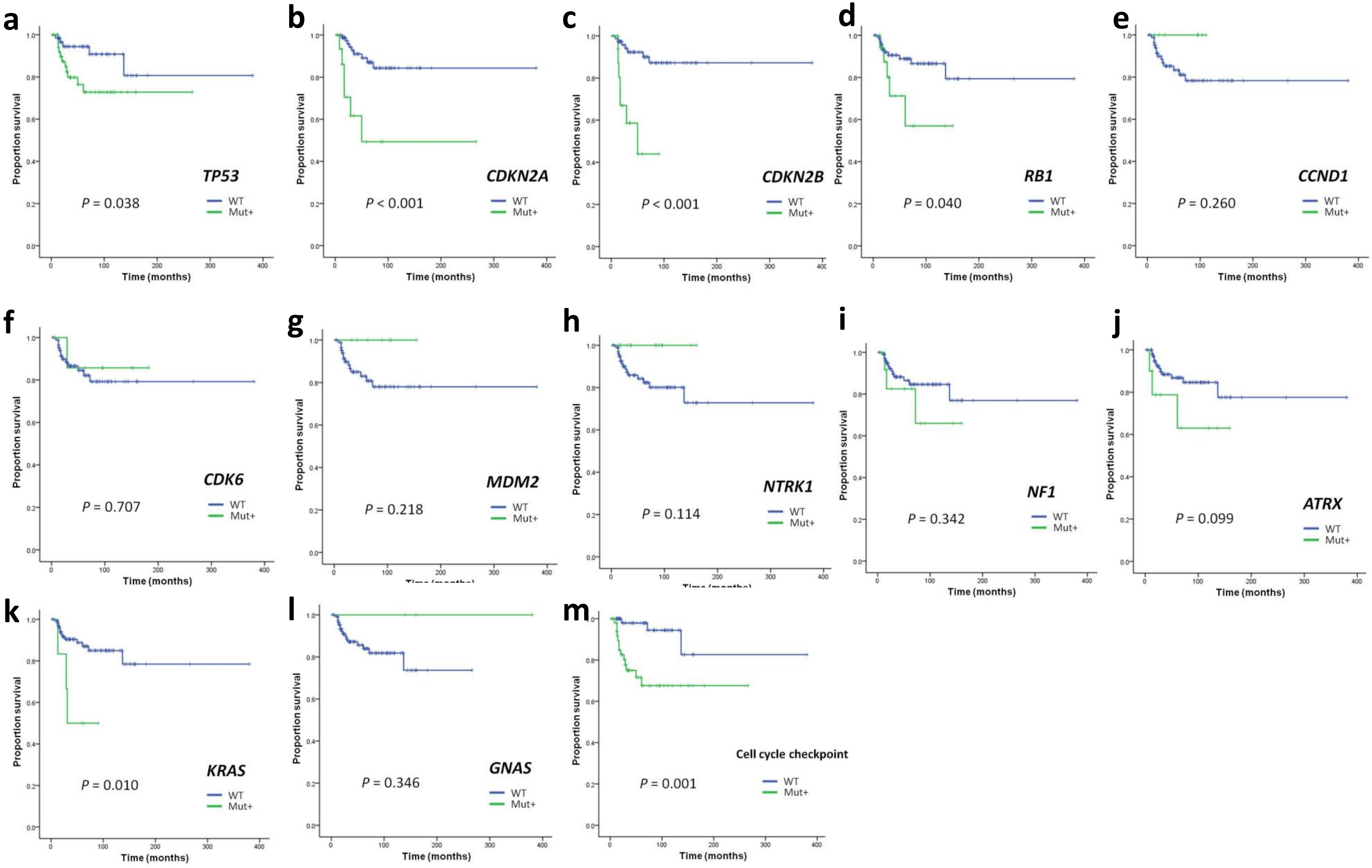

Supplementary Figure 4. Kaplan-Meier survival plots of overall survival stratified by absence or presence of gene alterations

TP53 (a), *CDKN2A* (b), *CDKN2B* (c), *RB1* (d), *CCND1* (e), *CDK6* (f), *MDM2* (g), *NTRK1* (h), *NF1* (i), *ATRX* (j), amplification of *KRAS* (k), somatic mutation of *GNAS* (l), and cell cycle checkpoints overall (m).

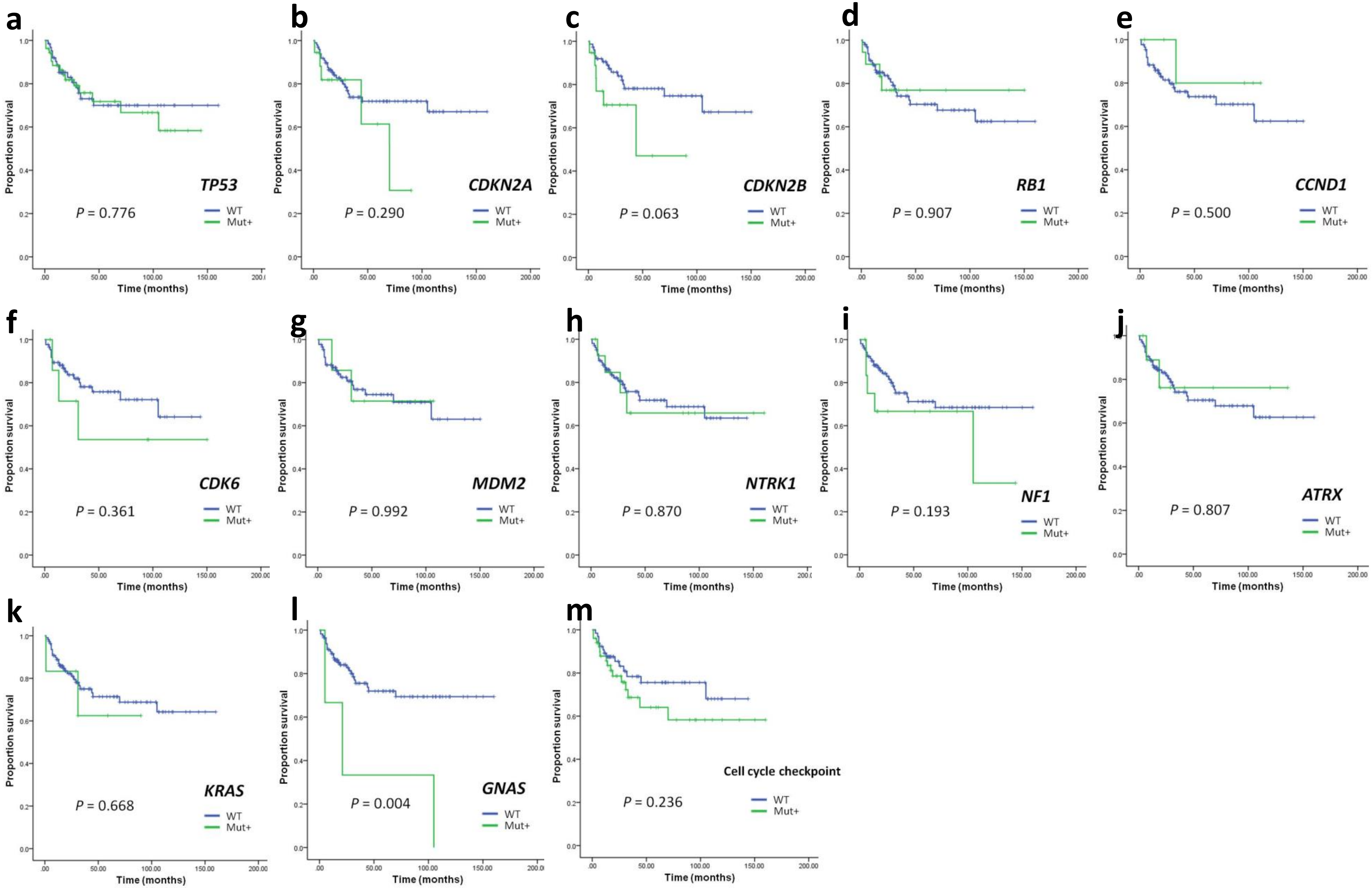

Supplementary Figure 5. Kaplan-Meier survival plots of local recurrence-free survival stratified by absence or presence of gene alterations

TP53 (a), *CDKN2A* (b), *CDKN2B* (c), *RB1* (d), *CCND1* (e), *CDK6* (f), *MDM2* (g), *NTRK1* (h), *NF1* (i), *ATRX* (j), amplification of *KRAS* (k), somatic mutation of *GNAS* (l), and cell cycle checkpoints overall (m).

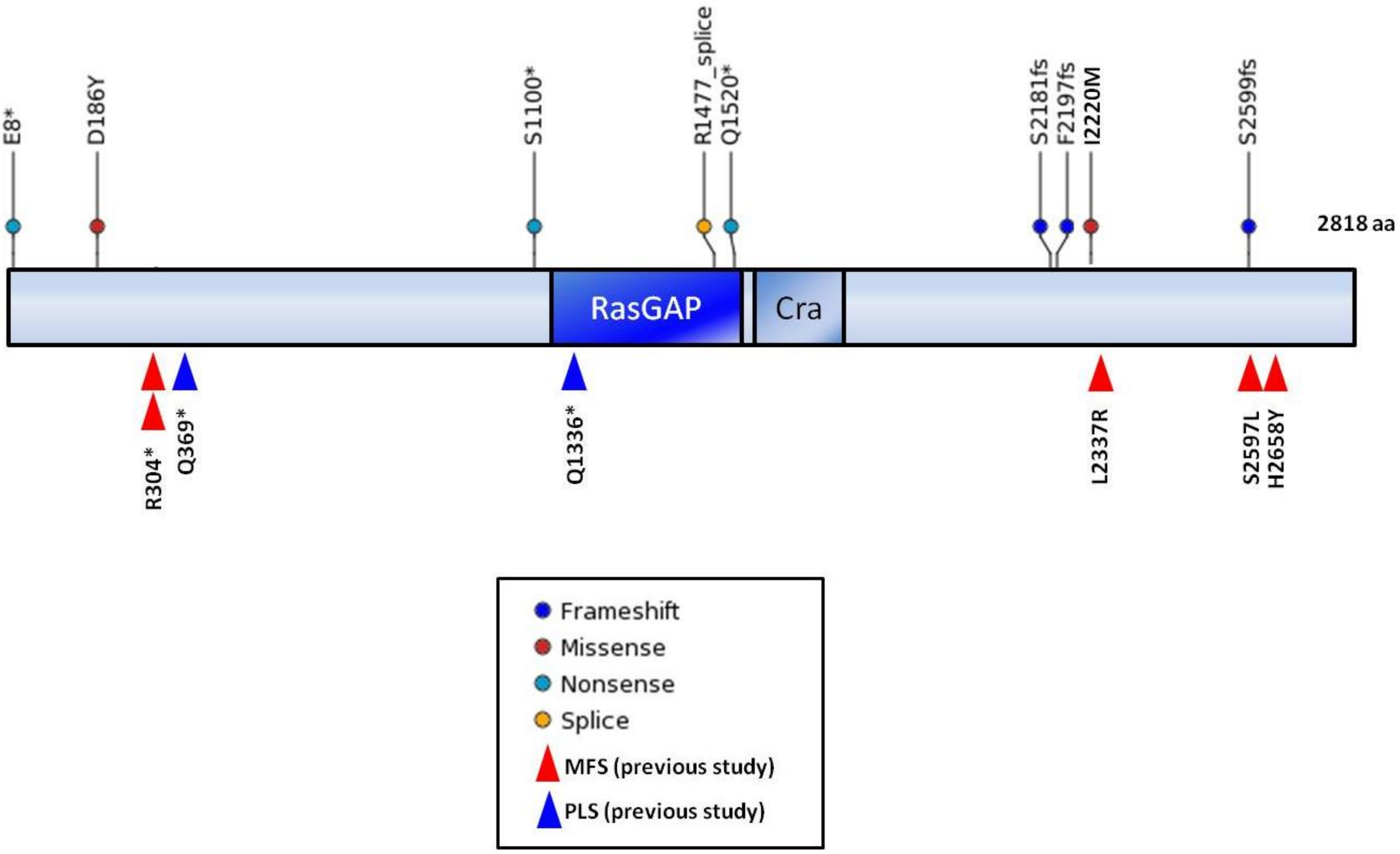

Supplementary Figure 6. NF1 mutations in the present and previous studies in MFS
The location of somatic mutations in NF1 observed in this study is indicated above a schematic of the NF1 protein, and those previously reported in MFS (blue triangles) and PLS (red triangles) are indicated below.
Types of mutations are indicated by color: frameshift indel, blue; missense, red; nonsense, light blue; and splice site, orange.

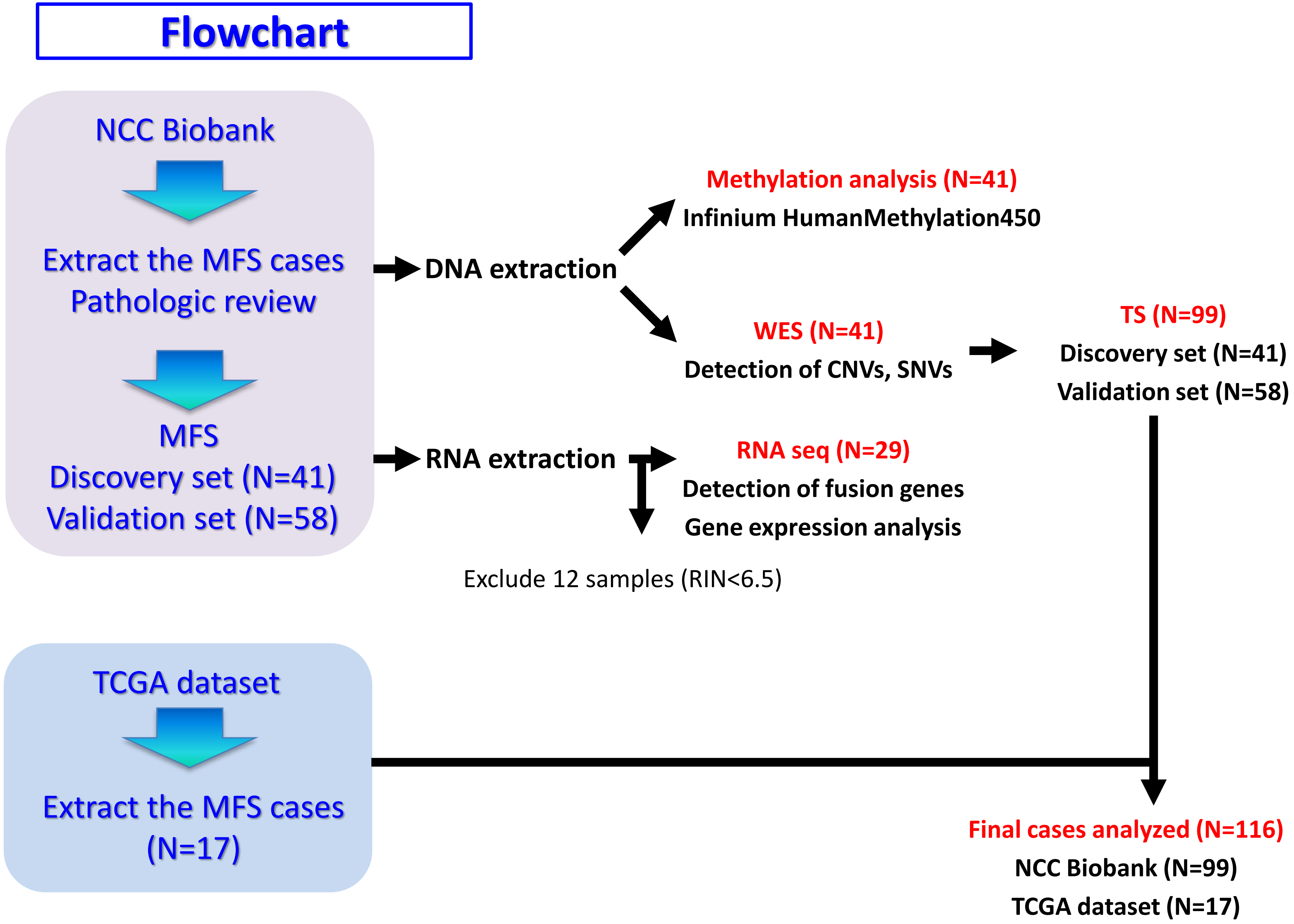

Supplementary Figure 7. Flowchart of the analysis

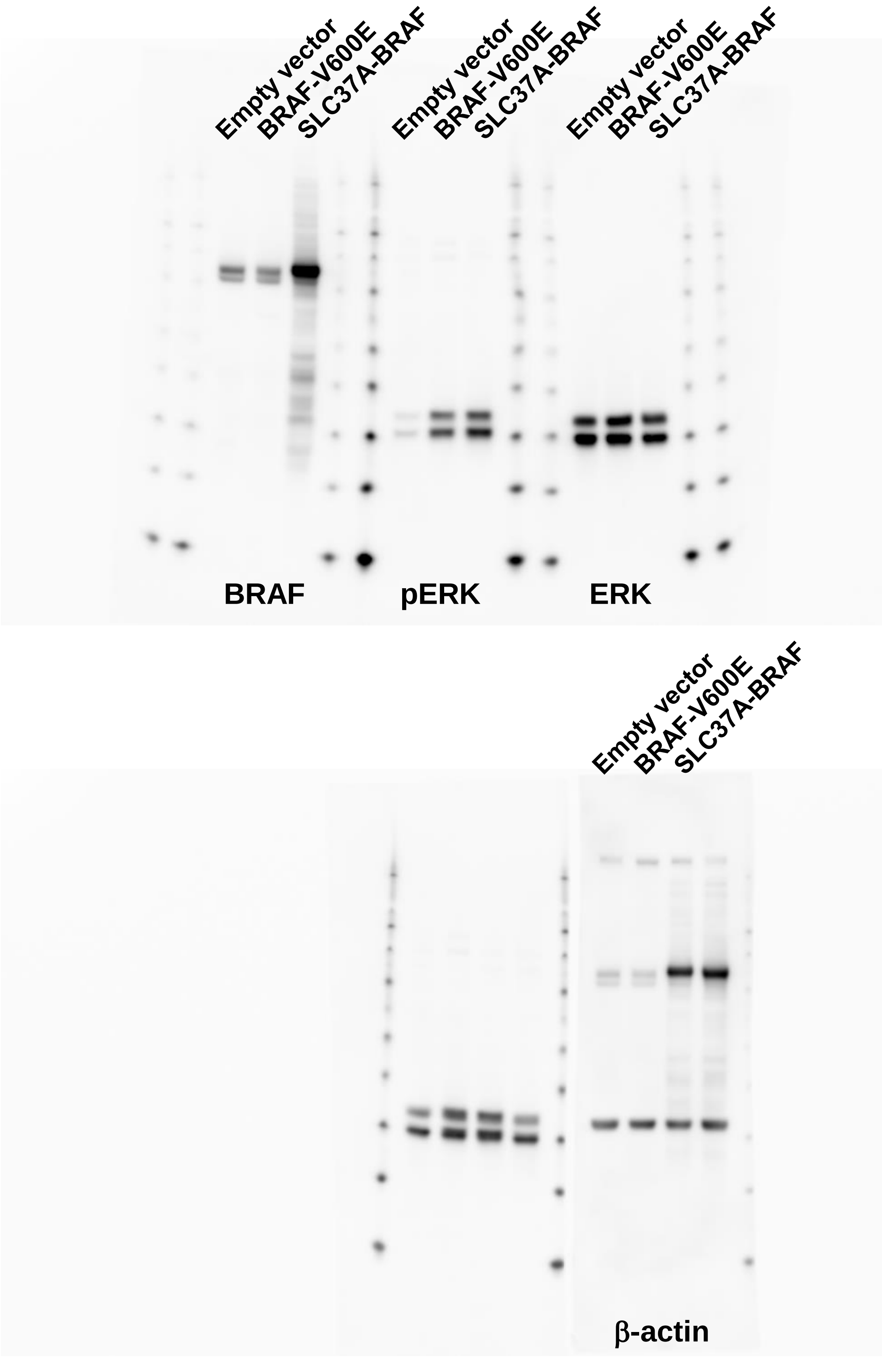

Supplementary Figure 8. Original images of the immunoblots shown in Fig. 3b

Supplementary Table 1. List of 140 genes included in the targeted capture sequencing

ABCA13	*CIC*	*FGFR2*	*HYDIN*	*MEP1A*	*NTRK1*	*S100A3*
AEN	*CLIP2*	*FGFR3*	*IDH1*	*MET*	*OR4X1*	*SAMSN1*
ALK	*CTNNB1*	*FGFR4*	*IDH2*	*MMEL1*	*PCDH19*	*SETD2*
AMACR	*DAXX*	*FLT3*	*INPPL1*	*MST1R*	*PDGFRA*	*SKP2*
ANK1	*DCC*	*FLT4*	*JAK1*	*MTAP*	*PDGFRB*	*SMARCA4*
ARID1A	*DNMT1*	*FOXA1*	*JAK2*	*MTHFR*	*PI4KA*	*SMARCB1*
ARID1B	*DNMT3A*	*FRYL*	*JAK3*	*MUC17*	*PIK3CA*	*STAT3*
ARID2	*EGFR*	*GAS8*	*KDM5C*	*MYC*	*PIK3R1*	*STK11*
ASS1	*EPHA1*	*GATA3*	*KDM6A*	*MYCL1*	*PIK3R3*	*SYK*
ATP1A3	*EPHA5*	*GNAS*	*KIAA1199*	*MYOCD*	*PLEKHA7*	*TDRD5*
ATR	*EPHA7*	*GPR64*	*KIAA1549*	*NACA*	*PML*	*TERT*
ATRX	*EPHA8*	*GRIA1*	*KIAA2022*	*NBPF1*	*POF1B*	*TET2*
BRAF	*ERBB2*	*GRIN2B*	*KIT*	*NF1*	*PRAMEF1*	*TIGD2*
CAMTA1	*ERBB3*	*H3F3A*	*KRAS*	*NKX2-1*	*PRDM1*	*TMPRSS11F*
CCND1	*ERBB4*	*H3F3B*	*LAMA4*	*NLRP9*	*PTEN*	*TNXB*
CDC73	*ERC2*	*HIST1H3B*	*LTK*	*NOTCH1*	*PTK2B*	*TP53*
CDH1	*EXT1*	*HOXA11*	*MAP2*	*NOTCH2*	*PTPRB*	*UNC80*
CDK6	*EZH2*	*HOXA13*	*MARVELD3*	*NOTCH3*	*RB1*	*WISP2*
CDKN2A	*FBXW7*	*HOXA9*	*MCYN*	*NPM1*	*RET*	*WT1*
CDKN2B	*FGFR1*	*HRAS*	*MDM2*	*NRAS*	*RTL1*	*ZCCHC6*

Promoter

These genes were chosen on the basis of the WES data of 41 MFSs (*p* -values) and the associated genes. Genes of interest from a review of the published literature were also chosen.

WES, whole-exome sequencing; MFS, myxofibrosarcoma.

Supplementary Table 2. Comparison of major alterations in various soft tissue sarcomas with complex karyotypes using our data and TCGA dataset (N = 290)

Altered gene	DDLS (N = 50), %	LMS (N = 80), %	MFS (N = 116), %	UPS (N = 44), %	*P*- value
TP53	4.0	61.3	45.7	63.6	<0.001
MDM2	94.0	0.0	6.0	0.0	<0.001
CDKN2A	6.0	7.5	15.5	18.2	0.104
CDKN2B	6.0	7.5	15.5	18.2	0.104
RB1	0.0	37.5	18.1	36.4	<0.001
CCND1	8.0	2.5	6.0	0.0	0.180
CDK6	0.0	0.0	6.9	9.1	0.014
CDK4	86.0	0.0	0.0	2.3	<0.001
NF1	8.0	3.8	11.2	4.5	0.218
PTEN	0.0	18.8	3.4	6.8	<0.001
KRAS	8.0	1.3	6.9	2.3	0.170
NTRK1	8.0	2.5	9.5	11.4	0.216
SYK	4.0	1.3	3.4	0.0	0.456
PIK3CA	0.0	2.5	0.9	6.8	0.076
GNAS	8.0	1.3	8.6	6.8	0.184
JAK1	14.0	3.8	4.3	4.5	0.063
FOXA1	0.0	1.3	6.9	4.5	0.089
NKX2-1	0.0	0.0	6.0	0.0	0.013
ATRX	34.0	16.3	8.6	31.8	<0.001
TET2	2.0	1.3	3.4	2.3	0.795
SETD2	0.0	2.5	2.6	4.5	0.551
FLT4	10.0	1.3	4.3	0.0	0.036
CDH1	0.0	0.0	2.6	0.0	0.208

Supplementary Table 3. Hazard ratio stratified by presence or absence of gene alteration (Cox proportional hazards model, univariate analysis)

Altered gene	Frequency (%)	Overall survival		Metastasis-free survival		Local recurrence-free survival	
		Hazard ratio (95% CI)	*P*- value	Hazard ratio (95% CI)	*P*- value	Hazard ratio (95% CI)	*P*- value
TP53	46%	2.92 (1.01–8.42)	0.048	1.38 (0.65–2.94)	0.405	1.11 (0.54–2.30)	0.777
RB1	18%	2.91 (1.00–8.42)	0.049	2.32 (0.98–5.52	0.056	0.94 (0.33–2.70)	0.907
CDKN2A	16%	10.85 (3.69–31.93)	<0.001	3.42 (1.43–8.17)	0.006	1.90 (0.69–5.24)	0.394
CDKN2B	16%	7.52 (2.51–22.50)	<0.001	3.06 (1.27–7.37)	0.012	2.41 (0.92–6.32)	0.073
NF1	11%	1.82 (0.52–6.40)	0.350	0.99 (0.30–3.30)	0.992	1.88 (0.72–4.93)	0.201
NTRK1	9%	0.04 (0.00–20.67)	0.312	2.12 (0.85–5.25)	0.106	1.09 (0.38–3.14)	0.870
GNAS	9%	0.04 (0.00–1044.34)	0.540	0.04 (0.00–185.69)	0.463	4.86 (1.46–16.19)	0.010
ATRX	9%	2.75 (0.78–9.66)	0.114	1.37 (0.41–4.57)	0.604	0.84 (0.20–3.52)	0.808
KRAS	7%	4.58 (1.29–16.30)	0.019	4.82 (1.65–14.06)	0.004	1.00 (1.00–1.00)	0.351
CDK6	7%	0.68 (0.09–5.21)	0.709	1.70 (0.50–5.75)	0.394	1.75 (0.52–5.92)	0.369
MDM2	6%	0.04 (0.00–99.91)	0.425	0.51 (0.07–3.78)	0.508	0.99 (0.23–4.26)	0.992
CCND1	6%	0.04 (0.00–189.51)	0.463	0.47 (0.06–3.47)	0.456	0.51 (0.07–3.79)	0.509

CI, confidence interval.
