# Supplementary material for: Integrated genetic and epigenetic analysis of myxofibrosarcoma
Source: Nat Commun. 2018 Jul 17;9:2765. doi: 10.1038/s41467-018-03891-9 (PMC6050269; doi:10.1038/s41467-018-03891-9)
Supplement: Supplementary file 3 — Description of Additional Supplementary Files [file 41467_2018_3891_MOESM3_ESM.pdf]

**Description of Additional Supplementary Files**

File Name: Supplementary Data 1
Description: Supplementary Data 1a. List of recurrently mutated genes identified by WES (Top 50, sorted by q-value (Geometrix Meean) and p-value (AMM)). Supplementary Data 1b. List of recurrently altered genes identified by WES (Top 50, sorted by q-value (Geometrix Meean) and p-value (AMM)).

File Name: Supplementary Data 2
Description: Supplementary Data 2a. Significant gain regions detected by GISTIC analysis. Supplementary Data 2b. Significant loss regions detected by GISTIC analysis.

File Name: Supplementary Data 3
Description: Fusion genes indentified in MFS.

File Name: Supplementary Data 4
Description: Significantly up-regulated genes among 29 MFS cases based on RNA sequencing data (sorted by p-value). Supplementary Data 4b. Significantly down-regulated genes among 29 MFS cases based on RNA sequencing data (sorted by p-value).

File Name: Supplementary Data 5
Description: Significant hypermethylated genes ($q < 0.2$).

File Name: Supplementary Data 6
Description: Supplementary Data 6a. Overrepresented annotation terms in Cluster B compared with Cluster A among significantly methylated genes. Supplementary Data 6b. Overrepresented annotation terms in Cluster C compared with Cluster A among significantly methylated genes. Supplementary Data 6c. Overrepresented annotation terms in Cluster C compared with Cluster B among significantly methylated genes.

File Name: Supplementary Data 7
Description: Genes with significantly correlation between methylation and expression data (sorted by p-value).

File Name: Supplementary Data 8
Description: Characteristics of patients with MFS (N = 116).
